# Development and Validation of a Path Length Calculation for Carotid–Femoral Pulse Wave Velocity Measurement: A TASCFORCE, SUMMIT, and Caerphilly Collaborative Venture

**DOI:** 10.1161/HYPERTENSIONAHA.117.10620

**Published:** 2018-04-11

**Authors:** Jonathan R. Weir-McCall, Liam Brown, Jennifer Summersgill, Piotr Talarczyk, Michael Bonnici-Mallia, Sook C. Chin, Faisel Khan, Allan D. Struthers, Frank Sullivan, Helen M. Colhoun, Angela C. Shore, Kunihiko Aizawa, Leif Groop, Jan Nilsson, John R. Cockcroft, Carmel M. McEniery, Ian B. Wilkinson, Yoav Ben-Shlomo, J. Graeme Houston

**Affiliations:** From the Division of Molecular and Clinical Medicine, College of Medicine, University of Dundee, United Kingdom (J.R.W.-M., L.B., J.S., F.K., A.D.S., J.G.H.); NHS Tayside Clinical Radiology, Ninewells Hospital, Dundee, United Kingdom (P.T., M.B.-M., S.C.C.); Department of Research and Innovation, North York General Hospital, University of Toronto, Canada (F.S.); Centre for Genomic and Experimental Medicine, The University of Edinburgh, Western General Hospital, United Kingdom (H.M.C.); NIHR Exeter Clinical Research Facility, Royal Devon and Exeter Hospital and University of Exeter Medical School, United Kingdom (A.C.S., K.A.); Lund University Diabetes Centre, Lund University, Malmö, Sweden (L.G.); Department of Clinical Sciences Malmö, Lund University, Sweden (J.N.); Department of Cardiology, Wales Heart Research Institute, Cardiff, United Kingdom (J.R.C.); Division of Experimental Medicine & Immunotherapeutics, University of Cambridge, United Kingdom (C.M.M., I.B.W.); and Population Health Sciences, University of Bristol, United Kingdom (Y.B.-S.).

**Keywords:** arteriosclerosis, atherosclerosis, cardiovascular diseases, hypertension, magnetic resonance angiography, pulse wave analysis

## Abstract

Current distance measurement techniques for pulse wave velocity (PWV) calculation are susceptible to intercenter variability. The aim of this study was to derive and validate a formula for this distance measurement. Based on carotid femoral distance in 1183 whole-body magnetic resonance angiograms, a formula was derived for calculating distance. This was compared with distance measurements in 128 whole-body magnetic resonance angiograms from a second study. The effects of recalculation of PWV using the new formula on association with risk factors, disease discrimination, and prediction of major adverse cardiovascular events were examined within 1242 participants from the multicenter SUMMIT study (Surrogate Markers of Micro- and Macrovascular Hard End-Points for Innovative Diabetes Tools) and 825 participants from the Caerphilly Prospective Study. The distance formula yielded a mean error of 7.8 mm (limits of agreement =−41.1 to 56.7 mm; *P*<0.001) compared with the second whole-body magnetic resonance angiogram group. Compared with an external distance measurement, the distance formula did not change associations between PWV and age, blood pressure, or creatinine (*P*<0.01) but did remove significant associations between PWV and body mass index (BMI). After accounting for differences in age, sex, and mean arterial pressure, intercenter differences in PWV persisted using the external distance measurement (*F*=4.6; *P*=0.004), whereas there was a loss of between center difference using the distance formula (*F*=1.4; *P*=0.24). PWV odds ratios for cardiovascular mortality remained the same using both the external distance measurement (1.14; 95% confidence interval, 1.06–1.24; *P*=0.001) and the distance formula (1.17; 95% confidence interval, 1.08–1.28; *P*<0.001). A population-derived automatic distance calculation for PWV obtained from routinely collected clinical information is accurate and removes intercenter measurement variability without impacting the diagnostic utility of carotid–femoral PWV.

## Abstract 2

Supplemental Digital Content is available in the text.

**See Editorial Commentary, pp 819–821**

Arteriosclerosis is the stiffening of the arterial wall, which occurs with advancing age and is strongly associated with risk of future cardiovascular events.^1^ Carotid–femoral pulse wave velocity (PWV) is the current gold standard for the assessment of aortic stiffness and has been included in guidelines on blood pressure management and as an end point in randomized clinical trials.^2,3^

Calculation of PWV requires measurement of both a time interval between the arrival of pressure waves at the carotid and femoral arteries and the distance between the 2 measurement sites. Despite the widespread use of PWV, there exists significant discrepancies in how the distance is measured because of the necessity of estimating the distance traveled by the tortuous arteries within the body which cannot be appreciated or quantified without imaging. Current recommendations advise for a direct distance to be used with a modifier of 0.8 based on a single study of 98 healthy volunteers who underwent magnetic resonance imaging (MRI).^4,5^ However, other studies of similar size using invasive measurements of PWV suggest a 2 measurement subtraction technique as the most accurate.^6^ Even using the direct measurement technique, it is widely recognized that normal reference values vary from center to center, suggesting either a real difference in PWV between geographical communities of healthy adults or that intercenter technique variability exists.^7^

A need therefore exists to create a more reproducible technique for the assessment of distance for pulse wave calculation to aid in the discrimination between health and disease and to improve applicability of published reference ranges and cutoff values for consideration of elevated risk. Whole-body magnetic resonance angiography (WB-MRA) allows the acquisition of the entire arterial tree in a single examination, allowing a direct arterial path length to be calculated. Using a suitably large data set, it is, thus, feasible that a formula for the calculation of path length based on routinely available metrics could be generated. The aim of this study was 4-fold: first to develop a formula for measuring arterial path length using only routine clinical data by training it against an MRI-based gold standard; second, to examine the performance of the formula in predicting the MRI-based path length in a validation data set; the third aim was to test whether use of this formula in 2 separate data sets from the training data set reduces intercenter differences in the distribution of PWV measurements from similar clinical groups; and finally, to test whether covariate associations with PWV using the new MRI learned path length formula differ from the associations using the current routinely used distance techniques in the 2 validation data sets. We hypothesized that a standardized formula for the assessment of path length would reduce intercenter variability without affecting the clinical application of the derived PWV.

## Methods

### Study Populations

Data from 3 study populations—the TASCFORCE study (Tayside Screening for Cardiovascular Events), the SUMMIT study (Surrogate Markers of Micro- and Macrovascular Hard End-Points for Innovative Diabetes Tools), and the CaPS (Caerphilly Prospective Study)—were used in the current study. The rationale, study design, techniques, and population demographics of each of these have been described in detail previously.^8–10^ These prior studies and the current study have been approved by an institutional review committee, with all subjects providing written informed consent. The study was performed in adherence with the principles of the Declaration of Helsinki. The TASCFORCE data that support the findings of this study are available from the corresponding author on reasonable request. The SUMMIT data are available from H.M. Colhoun, A.C. Shore, L. Groop, and J. Nilsson on reasonable request. The CaPS data are available from Y. Ben-Shlomo on reasonable request.

The TASCFORCE study is a population study comprised of low to intermediate risk participants free from clinically apparent cardiovascular disease who underwent WB-MRA from whom the path length formula was generated. The SUMMIT study is a multicenter European study examining markers of cardiovascular risk in a mixed population inclusive of those with and without diabetes mellitus and with and without established cardiovascular disease. All PWV measurements were performed using a SphygmoCor device, using a 4-point distance technique using the suprasternal notch to umbilicus distance plus the umbilicus to the femoral distance minus the carotid to suprasternal notch distance, henceforth, referred to as PWV_SUMM_. One hundred fifty-eight SUMMIT study participants also underwent WB-MRA using the same technique as those in TASCFORCE as part of a related substudy.^11^ CaPS is a population-based cohort study which tried to recruit all men aged 45 to 59 years residing in the town of Caerphilly between 1979 and 1983. Some of these men underwent PWV measurement at the fifth follow-up. In this populace, distance was measured using a 3-point technique measuring from the suprasternal notch to the femoral artery, minus the carotid to suprasternal notch distance, henceforth, referred to as PWV_CaPS_. In the SUMMIT substudy, the arterial path length formula generated from the TASCFORCE WB-MRA was compared against the same measurements obtained using the same WB-MRA technique in a separate population. In the main SUMMIT study, the effects of the formula on intercenter PWV variability were examined as were its effects on the association between PWV and cardiovascular risk factors. CaPS data were used to examine if these effects are replicated in a population in whom PWV has been calculated using a different distance measurement formula and its effects on the prognostic capability of PWV.

### Path Length Formula

**Figure 1.**
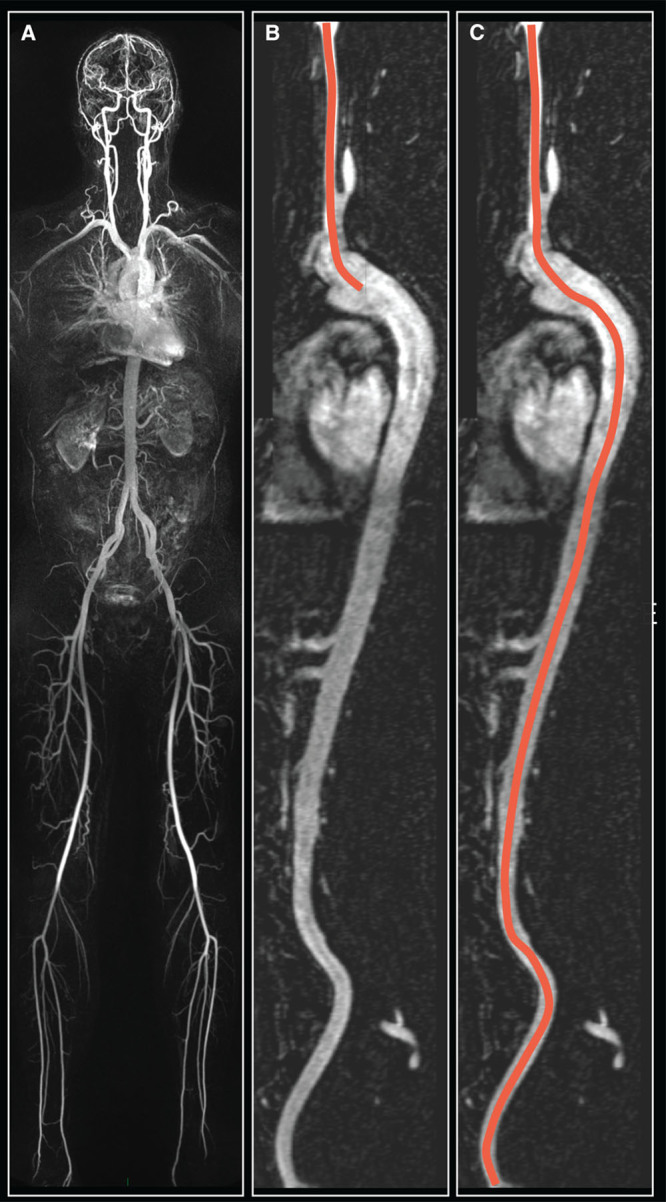
Demonstration of calculation of the right carotid femoral intra-arterial path length. Initially a whole-body magnetic resonance angiogram is produced (**A**), with a curved multiplanar reformat generated starting at the bifurcation of the right common carotid artery extending to the bifurcation of the right common femoral artery (**B** and **C**). The proximal distance from the common carotid bifurcation to the aorta is measured (**B**), with the common carotid to common femoral distance then measured (**C**). The path length is then calculated as C–2×B.

For the generation of the path length formula, the TASCFORCE study in which 1528 study participants underwent WB-MRA was used. The WB-MRA acquisition has been described in detail previously.^12^ To generate the path lengths, the 3D WB-MRA data sets were viewed offline (Carestream PACS Client Suite Version 10.1 sp1, Rochester, NY) by 1 of 4 image analysts. An arterial centerline was drawn between the bifurcation of the right common carotid artery and the right common femoral artery. From this a curved multiplanar reformat of the vessel was generated. The distance from the carotid bifurcation to the aortic arch was measured (proximal distance) and the distance from the aortic arch to the common femoral artery bifurcation (distal distance) was measured (see Figure 1). The distances were measured twice and an average of the 2 was used for subsequent analysis. For generation of the formula, the proximal distance was subtracted from the distal distance to represent the distance traveled by the pulse wave. Interobserver variability was assessed in 50 cases to ensure inter-reader consistency. The formula for the calculation of the distance was then generated using backward linear regression (see below for details of this). Although the left carotid–femoral distance was also measured and a formula generated, none of the clinical studies recorded side measured, and as the right side is recommended for measurement of PWV when possible,^13^ we have thus focused on this for the current study. The distance formula was used to recalculate PWV by using the formula distance divided by the carotid–femoral time interval produced by the SphygmoCor devices. For the remainder of the article, the PWV calculated using the WB-MRA derived formula distance shall be referred to as PWV_MRA_.

### External Validation

One hundred forty-eight participants of the SUMMIT study underwent WB-MRA using the same acquisition parameters and with the intra-arterial path length measured using the same technique as in the TASCFORCE cohort.^14^ This was then compared with the distance generated using the formula from the TASCFORCE cohort. PWV in this cohort was calculated using the 3 distances: the external distance (PWV_SUMM_), the true distance on MRI (PWV_TD_), and the formula distance (PWV_MRA_).

### External Application

Using the formula, PWV_MRA_ was calculated in both the SUMMIT and CaPS population. This was then compared with the PWV_SUMM_ in the SUMMIT cohort for its effects on intercenter variability and the associations between PWV and common risk factors and the PWV_CaPS_ in the CaPS population for its effects on the association between PWV and risk factors.

### Statistical Analysis

Data are expressed as mean±SD for continuous variables, median (range) for ordinal variables, and N (%) for nominal variables. Normality tests with Shapiro–Wilk were undertaken. For derivation of a path length formula within the TASCFORCE population, the influence of clinical factors on carotid–femoral path length was quantified using linear regression modeling. Sex, age, height, weight, waist circumference, systolic blood pressure, diastolic blood pressure, heart rate, cholesterol, high-density lipoprotein, low-density lipoprotein, triglycerides, glucose, smoking status, smoking years, pack years, and BMI were the starting variables within the backward entry model. To avoid overfitting, the variables were initially split into 2 blocks and backward linear regression preformed. The variables remaining in the model at the end of the backward linear regression then inserted into a third backward linear regression performed to generate the final distance formula. This formula was compared with the path length measured on MRI in the SUMMIT substudy using Bland–Altman plots and 2-way mixed absolute agreement intraclass correlation coefficient. Association between PWV and clinical variables was assessed using multiple linear regression with age, sex, and mean arterial pressure (MAP) included as covariates in all regressions. Analysis of variance was used for the comparison of means when ≥3 groups were compared. Paired *t* tests were used to compare the PWV measurements obtained using the derived formula and the original physical distance measurement. For comparing the PWV between centers, Analysis of covariance was run with PWV as an independent variable, study center as a fixed variable, and age, sex, and MAP as covariates. As only 2 of the 3 SUMMIT centers recruited those under the age of 62 years and because of the known strong confounding effect of age, comparison between the 3 SUMMIT sites was limited to participants >62 years of age. The CaPS cohort only involved males ≥65 years, with a significantly lower prevalence of cardiovascular disease than SUMMIT. As all these factors are known to influence PWV considerably, only males ≥65 years of age and free from cardiovascular disease were included in the analysis comparing the PWV in the 4 centers (Dundee, Exeter, Lund, and Caerphilly). Logistic regression analyses were undertaken to look at associations with cardiovascular outcomes. All data were analyzed using SPSS statistical package (version 21.0, IBM SPSS, Chicago, IL) and RStudio (The R Foundation, Version 3.3.2). Significance was assumed when *P*<0.05.

## Results

### Study Populations

The demographics of the 3 different studies are described in Table 1.

**Table 1.**
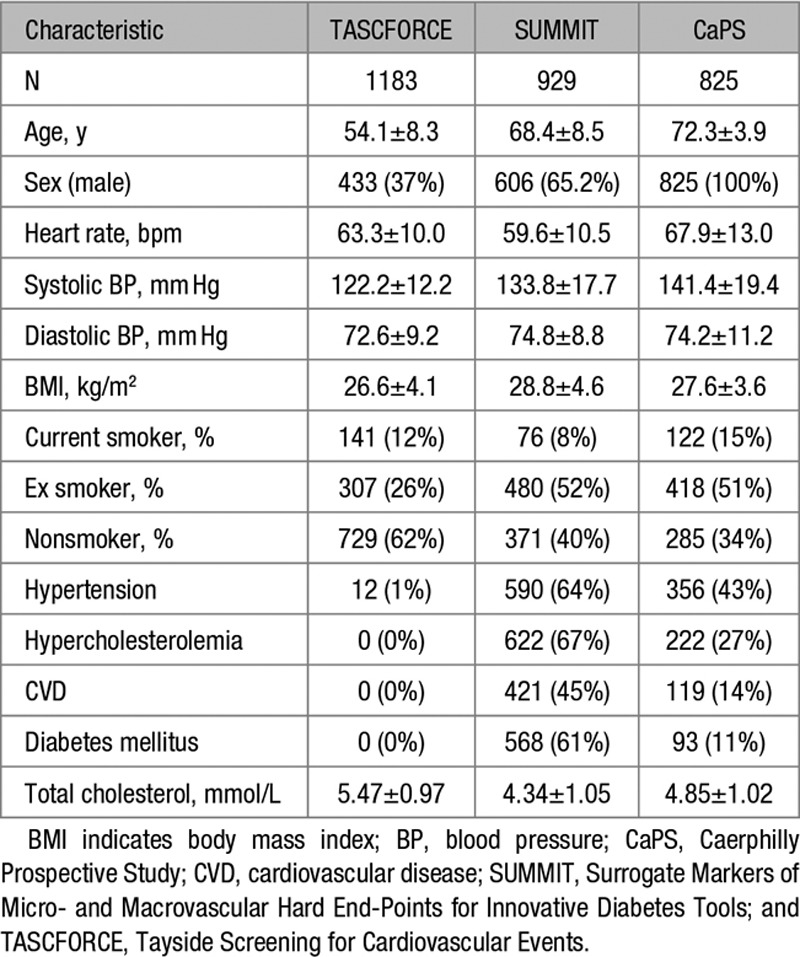
Characteristics of the 3 Study Cohorts

### Path Length Formula

The number of WB-MRA subjects available for analysis were 1513. Of these, 240 were discarded as one of the readers for path length measurement provided significantly greater distances than the other 3 readers on the interobserver analysis. Ninety additional data sets were discarded because of technical issues in creating the whole-body angiograms from the 4 separate angiography sequences. This left 1183 data sets for generation of the formula. On backwards linear regression, age, sex, heart rate, height, and weight were the strongest predictors of arterial path length, leaving the final formula for the calculation of the right sided distance being:


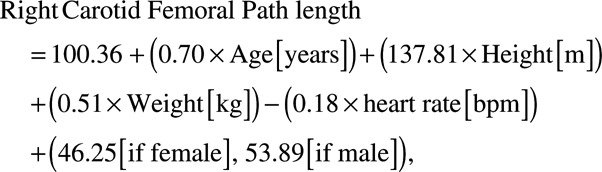
(1)


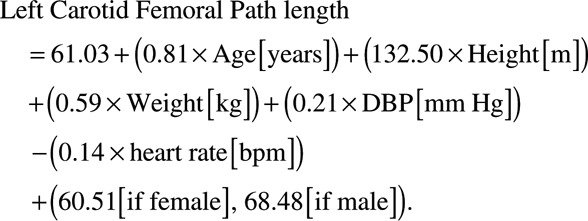
(2)

**Figure 2.**
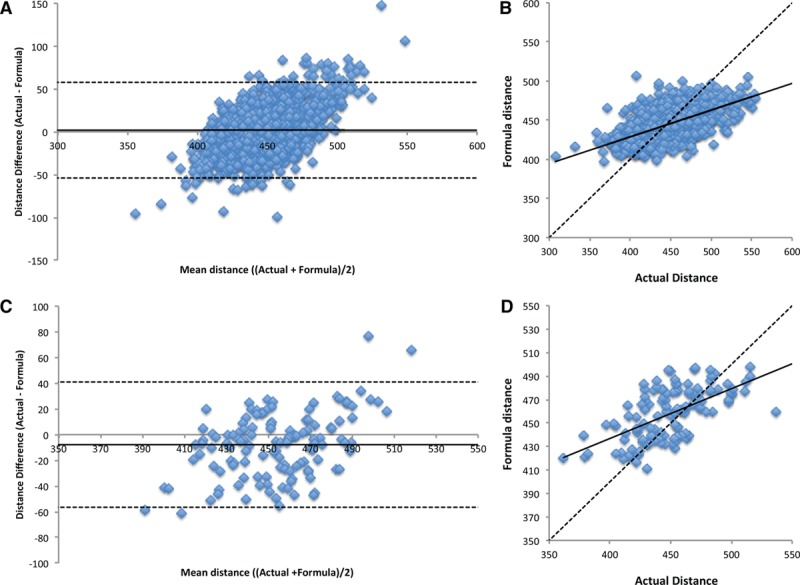
Bland–Altman and scatter plots of the predicted path length generated using the formula versus the true distance measured on whole body angiography in the TASCFORCE (**A** and **B**) and SUMMIT (**C** and **D**) cohorts. SUMMIT indicates Surrogate Markers of Micro- and Macrovascular Hard End-Points for Innovative Diabetes Tools; and TASCFORCE, Tayside Screening for Cardiovascular Events.

When applied within the TASCFORCE cohort, the formula produced a mean path length of 444.7±20.5 mm with the true distance with a true path length of 446±35 mm. There was a slight overestimate with a mean difference of 1.97 mm (limits of agreement =−53.9 to 57.8 mm; *P*=0.02), intraclass correlation coefficient =0.51 (95% confidence interval [CI], 0.47–0.55; *P*<0.001). There was some evidence of systematic bias, such that the formula overestimated short path lengths and underestimated long path lengths (see Figure 2A). Mean difference did not differ by age, but those over 70 years of age had wider variation between true distance and formula distance (Figure S1A in the online-only Data Supplement).

### External Validation

**Figure 3.**
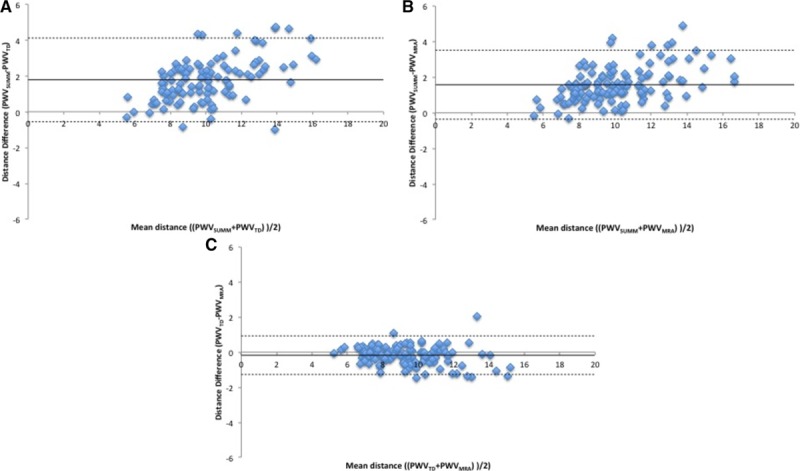
Bland–Altman plots comparing pulse wave velocity (PWV) calculated using the external distance (PWV_SUMM_), true distance on MRI (PWV_TD_), and formula distance (PWV_MRA_). **A**, PWV_SUMM_ compared with PWV_TD_; **B**, PWV_SUMM_ compared with PWV_MRA_; **C**, PWV_MRA_ compared with PWV_TD_. MRA indicates magnetic resonance angiography; MRI, magnetic resonance imaging; SUMMIT, Surrogate Markers of Micro- and Macrovascular Hard End-Points for Innovative Diabetes Tools; and TD, true distance.

Of the 148 SUMMIT WB-MRA data sets, a path length measurement was possible in 128, with 20 lost because of technical issues combining the 4 angiogram stations into a single whole-body data set. Measured distance in the cohort was 450±31 mm, compared with a formula distance of 458±22 mm. Compared with the measured intra-arterial path length on the MRI, there was a small but significant overestimation of the path length distance by the formula with a mean difference of 7.8 mm (limits of agreement =−41.1 to 56.7 mm; *P*<0.001) and a moderate correlation between the 2 measures with an intraclass correlation coefficient of 0.55 (95% CI, 0.41–0.67; *P*<0.001); see Figure 2B. Mean difference did not differ by age, but those over 70 years of age had wider variation between true distance and formula distance (Figure S1B). Compared with the PWV_TD_ (9.2±2.0 ms^−1^), PWV_SUMM_ (11.0±2.6 ms^−1^) was significantly higher with a mean difference of 1.78 ms^−1^ (limits of agreement =−0.56 to 4.12 ms^−1^; *P*<0.001), with a marked reduction in the magnitude of the difference and the variation when compared with PWV_MRA_ (9.37±2.2 ms^−1^), with a mean difference of −0.17 ms^−1^, although this remained statistically significant (limits of agreement =−0.56 to 0.28 ms^−1^; *P*=0.002); see Figure 3.

### External Application: SUMMIT

One thousand two hundred forty-two individuals underwent PWV measurement as part of the multicenter SUMMIT study. Three hundred forty-four individuals were excluded from analysis because of the center at which they were performed, not recording the distance and time components for PWV calculation in their metadata. This left with 1025 individuals, of whom 96 were excluded because of missing ≥1 of the variables required by the formula for the calculation of path length distance, leaving 929 in the final analysis (Table 1). PWV_SUMM_ in the cohort was 10.84±2.8 ms^−1^. When this was recalculated, there was a significantly lower PWV_MRA_ of 9.94±2.5 ms^−1^ (mean diff =0.90, SD=1.08, *P*<0.001). Compared with PWV_SUMM_, PWV_MRA_ demonstrated similar correlations with age, MAP, and HbA1c, and a loss of correlation with BMI, cholesterol, and with only a weak relation remaining between PWV_MRA_ and waist circumference and creatinine (see Table 3). Use of the distance formula did not weaken the difference in PWV between those with and those without cardiovascular disease (CVD), with significant differences observed using both the traditional PWV_SUMM_ technique (CVD positive =11.2±3.0 ms^−1^, CVD negative =10.5±2.5 ms^−1^, *t* =−3.6; *P*<0.001) and the PWV_MRA_ technique (CVD positive =10.3±2.7 ms^−1^, CVD negative =9.6±2.3 ms^−1^, *t*=−4.1; *P*<0.001). When comparing the PWV at the 3 European sites, there was a significant difference between all 3 using both PWV_SUMM_ (*F* test =19.4; *P*<0.001) and PWV_MRA_ (*F* test =17.4; *P*<0.001); however, there were differences in baseline variables between the 3 centers (see Table S1 for comparison of the baseline demographics between the 3 centers). After correcting for differences between centers in the distribution of important determinants of PWV—that is, age, sex, and MAP which are the strongest determinants for PWV—there remained a significant difference between sites for PWV_SUMM_ (*F* test =24.4; *P*<0.001), whereas the difference using PWV_MRA_ significantly reduced (*F*=5.0; *P*=0.007; see Table 2). Further corrections for BMI differences between centers did not result in any change in this outcome.

**Table 2.**
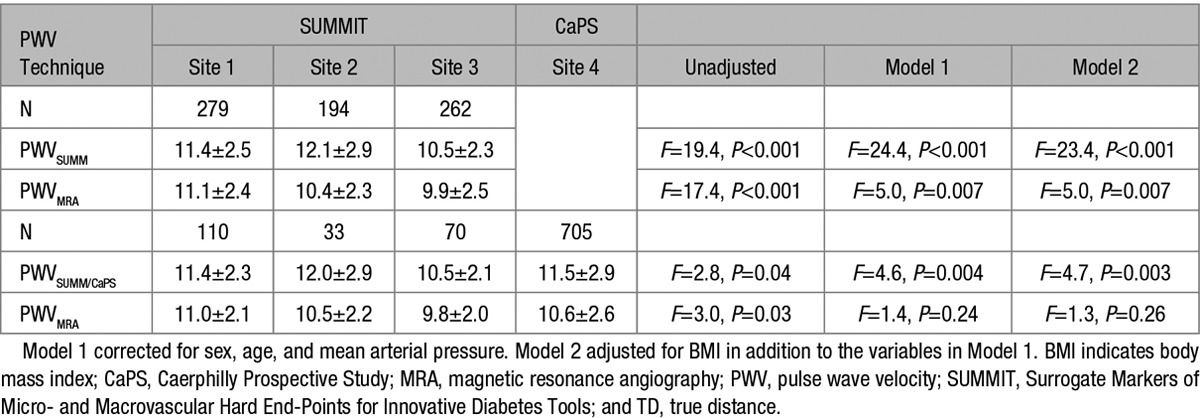
Comparison of PWV Across the 3 Sites Based on External Distance Measurement and Formula Distance Calculation in the SUMMIT Cohort and Between the 4 Sites When the CaPS Data Are Included

**Table 3.**
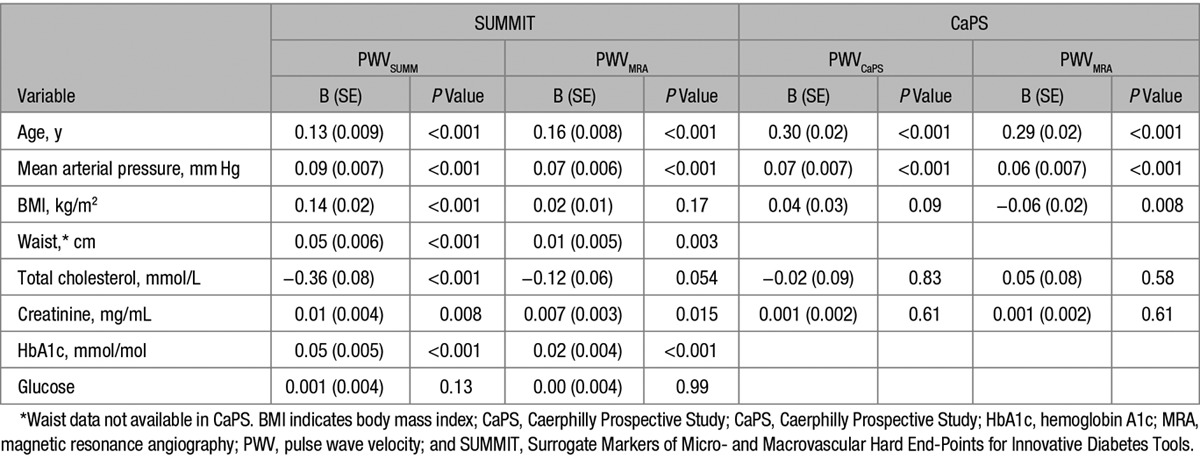
Association of PWV Based on External Distance Measurement and Formula Distance Calculation in the SUMMIT and CaPS Cohort

### External Application: CaPS

Eight hundred twenty-five males underwent PWV assessment at visit V. PWV using the external distance measurement was 11.6±2.9 ms^−1^. When this was recalculated, there was a significantly lower PWV of 10.7±2.7 ms^−1^ (mean difference =0.87, SD=0.93; *P*<0.001), although the magnitude of the difference was less pronounced than in the SUMMIT cohort. Association with age, MAP, and creatinine remained unchanged using either of the 2 distance techniques for PWV; however, the BMI showed a continued significant but inverse association with PWV_MRA_ (see Table 3).

At a median follow-up of 8.5 years (interquartile range, 4.6–9.2 years), 154 (18.7%) participants had suffered a major adverse cardiovascular event, of which 79 (9.6%) were fatal. The age- and systolic blood pressure–adjusted odds ratio for PWV_CaPS_ was 1.11 (95% CI, 1.04–1.18; *P*=0.003) per SD increase in PWV_CaPS_ for major adverse cardiovascular events and 1.14 (95% CI, 1.06–1.24; *P*=0.001) for fatal cardiovascular events using multivariable logistic regression. These results were almost unchanged when the analysis was repeated using PWV_MRA_, with the age- and systolic blood pressure–adjusted odds ratio being 1.11 (95% CI, 1.03–1.20; *P*=0.004) per SD increase in PWV_MRA_ for major adverse cardiovascular events and 1.17 (95% CI, 1.08–1.28; *P*<0.001) for fatal cardiovascular events.

## Discussion

In the current study, we have developed a formula for the calculation of interarterial distance for PWV measurement and applied this in 2 independent study populations across 4 European centers. Using this formula strengthens the association of PWV with traditional risk factors and removes differences between centers but does not reduce discrimination between those with and without cardiovascular disease, nor does it reduce the prognostic strength of PWV.

Distance measurement inaccuracies are an inherent source of error in PWV calculation. Multiple studies have documented significant differences between carotid–femoral PWV and central aortic PWV,^15,16^ with this difference being markedly reduced when the true arterial path lengths are used in the carotid–femoral PWV calculation rather than the external distance measurements.^14,17^ We are not the first to attempt to derive a formula for the calculation of the true arterial path length for use in PWV calculation. Weber et al^6^ produced a calculation for distance being equal to the (height/4+7.28) from a population of 135 being investigated for coronary artery disease, but, however, used the distance from the ascending aorta to the aortic bifurcation as the comparator which is not representative of the path traveled by the pulse wave between the carotid and femoral artery. Filipovsky et al^18^ derived the calculation (height×0.29) in a population of 596 individuals predominantly free from cardiovascular disease but used an externally measured distance as the gold standard with all the aforementioned weaknesses this entails. Both techniques were examined by Huybrechts et al^4^ in a population of 98 healthy men and women, which found that the Weber calculation was accurate but less so than a direct distance with a modifier of 0.8 and the Filipovsky calculation to be highly inaccurate. In our study, we found the distance formula to vary from the true distance by an average of 7.8±25 mm in an external validation cohort, which while having a minimally higher bias compared with the 2.6±38 mm reported for the direct distance×0.8 has far tighter limits of agreement. Although we observed significant variation between the formula and true path length of all variations were up to 100 mm in a small number of individuals within both the derivation and the validation cohort, this is likely a consequence of anatomic variability of the vascular tree rather than the formula, with differences in excess of 100 mm also reported between external distance measurements and true path length in previous studies.^4,6^ There also exists further potential for greater variability in the real world using the direct distance×0.8 technique, where choice of measurement device is also known to result in significantly different distances, with a sliding caliper producing shorter distances than tape measures, with this effect most pronounced in the obese.^13,19–21^

In the formula generated for the calculation of path length, we found height, weight, and heart rate to explain right carotid femoral path length. Although height and weight are well documented associates of body surface area and, therefore, vascular volume,^22^ it is not immediately apparent how heart rate would affect path length. One possibility is that as heart rate increases, stroke volume and, therefore, vascular distension falls, or alternately it may be that an increased heart rate reflects greater sympathetic activation with associated vascular constriction.^23^ As a result, if all other variables were to remain equal but heart rate were to change, the formula distance would shift. Further work is therefore required to determine the effects of the formula on variability of measurement of longitudinal changes.

The strengths of the current technique are its use of readily obtainable metrics, with age, height, and weight being almost ubiquitously collected in the routine clinical work-up of cardiovascular disease. Although the formula would be cumbersome if it had to be calculated by the clinician at the bedside, the calculation is far simpler than the multitude of calculations being undertaken by the devices used for PWV measurement currently, and if such an inbuilt calculation were to be demanded by the end point users, industry will almost certainly be quick to respond. By removing a further source of human error and variability, such a calculation will standardize measurements across the globe, improving the applicability of reference ranges and cutoff values used in guidelines for determining elevated risk.

In our study, as in previous work, we found a significant difference in PWV between centers even after accounting for age, sex, and MAP.^7^ In comparison, we found that using the formula generated in the TASCFORCE population, this difference was significantly reduced. Although this could be ascribed to regressing the PWVs of the study participants to a common mean, this would also reduce the association between risk factors and PWV and reduce the differences between those with and without disease. This was not the case in the current study with the only variables significantly impacted by the new formula being BMI and waist circumference—both of which are known to affect the accurate path length measurement.^14,20^ We found no significant shift in the magnitude of differences in PWV between those with and those without cardiovascular disease and no substantial change in the hazard ratios for PWV for prediction of future major adverse cardiovascular events and mortality.

Despite the robustness of our population derivation and external validation in multiple centers using different techniques, there remain some limitations within our study and a need for several avenues of further work. The formula was derived in a population of adults over 40 years of age and validated in populations with mean ages of 68 and 72 years; thus, the robustness of the distance formula in those under 40 years of age requires further study. All our centers were of European origin with 3 of the 5 populations examined being of predominantly white British descent, limiting conclusions to this populace. This is particularly pertinent given the reported differences in the prevalence of the different anatomic variations in the branching patterns of the aortic arch between populations, which could introduce a consistent bias.^24,25^ However, because the most common variant is a bovine arch which would affect left-sided distances rather than right and other variations are much less common, the impact of this is likely to be small and would reinforce the current guidelines of using right-sided distances whenever technically feasible. For our external validation, we assumed the distance to have been measured on the right hand side as per current recommendations because this was not recorded in the final study data, which will not have been the case in all participants. However, this would have confounded the benefit of the formula, and thus, its impact would be to cause an apparent worsening of its accuracy and correlations potentially underestimating its true benefits. Although we have examined the effects of the formula on correlation with risk factors, on intercenter variability, and on prognosis, its effects on net reclassification of risk has yet to be examined. Finally, while we examined it in comparison to 2 external distance measurement techniques, both of these techniques used were multipoint subtraction techniques rather than the direct carotid–femoral distance with a modifier of 0.8.^5^

## Perspectives

A population-derived automatic distance calculation for PWV obtained from routinely collected clinical information is accurate and reduces measurement variability without impacting the diagnostic utility of carotid–femoral PWV. Uptake of this will lead to improved technique standardization and applicability of guidelines and reference ranges.

## Sources of Funding

The TASCFORCE study (Tayside Screening for Cardiovascular Events) was funded by the Souter Charitable Foundation and the Chest, Heart and Stroke Scotland Charity. The SUMMIT study (Surrogate Markers of Micro- and Macrovascular Hard End-Points for Innovative Diabetes Tools) was supported by the Innovative Medicines Initiative (the SUMMIT consortium, IMI-2008/115006). The initial stages of the CaPS (Caerphilly Prospective Study) was funded by the MRC with a grant from the British Heart Foundation funding the measurement of the pulse wave velocity. The statistician was funded by TENOVUS, Tayside. J.R. Weir-McCall is supported by the Wellcome Trust through the Scottish Translational Medicine and Therapeutics Initiative (Grant no. WT 085664) in the form of a Clinical Research Fellowship. C.M. McEniery is supported by the NIHR (National Institute of Health Research) Cambridge Biomedical Research Centre. These groups did not have any role in study design; the collection, analysis, and interpretation of data; in the writing of the manuscript; nor in the decision to submit the manuscript for publication.

## Disclosures

None.

## Supplementary Material

## Novelty and Significance

### What Is New?

- We describe a formula to calculate pulse wave velocity (PWV) path length based on routinely available clinical information derived from 1183 whole-body magnetic resonance imagings.
- This formula reduces intercenter differences in PWV while retaining associations with risk factors, presence of cardiovascular disease, and risk prediction.

### What Is Relevant?

- Reduction of intercenter differences in PWV calculation which are known to exist will improve applicability of reference ranges.

### Summary

A population-derived automatic distance calculation for PWV obtained from routinely collected clinical information is accurate and removes intercenter measurement variability without impacting the diagnostic utility of carotid–femoral PWV.
