# Supplementary material for: Development and Validation of a Path Length Calculation for Carotid–Femoral Pulse Wave Velocity Measurement: A TASCFORCE, SUMMIT, and Caerphilly Collaborative Venture
Source: Hypertension. 2018 Apr 11;71(5):937–45. doi: 10.1161/HYPERTENSIONAHA.117.10620 (PMC5902134; doi:10.1161/HYPERTENSIONAHA.117.10620)
Supplement: Supplementary file 1 [file hyp-71-937-s001.docx]

**ONLINE SUPPLEMENT**

**Development and validation of a pathlength calculation for carotid-femoral pulse wave velocity measurement: A TASCFORCE, SUMMIT and Caerphilly collaborative venture**

Jonathan R Weir-McCall, Liam K Brown, Jennifer Summersgill, Piotr Talarczyk, Michael Bonnici-Mallia, Sook C Chin, Faisel Khan, Allan D Struthers, Frank Sullivan, Helen M Colhoun, Angela C Shore, Kunihiko Aizawa, Leif Groop, Jan Nilsson, John R Cockcroft, Carmel M McEniery, Ian B Wilkinson, Yoav Ben-Shlomo, J Graeme Houston.

**Corresponding author:** J Graeme Houston

Address: Division of Clinical and Molecular Medicine, Level 7, Ninewells Hospital, Dundee DD1 9SY, UK

Telephone: +44(0)1382-383468

E-mail: j.g.houston@dundee.ac.uk

**Table S1:** Comparison of baseline measures between the 3 SUMMIT sites in those ≥62 years old.

| **Characteristic** | **Site 1** | **Site 2** | **Site 3** | **P** |
| --- | --- | --- | --- | --- |
| N | 281 | 292 | 356 |  |
| Age (years) | 73.8 ± 5.1 | 64.8 ± 8.2 | 67.1 ± 8.7 | <0.001 |
| Sex (male) | 202 | 124 | 196 | 0.01 |
| Heart rate (bpm) | 57.4 ± 9.4 | 61.3 ± 11.9 | 59.9 ± 9.9 | 0.006 |
| Systolic BP (mmHg) | 138.8 ± 18.1 | 134.6 ± 17.9 | 129.2 ± 16.0 | <0.001 |
| Diastolic BP (mmHg) | 74.4 ± 8.9 | 77.2 ± 8.9 | 73.1 ± 8.1 | <0.001 |
| BMI (kg/m^2^) | 27.7 ± 3.9 | 29.0 ± 4.3 | 28.5 ± 4.1 | 0.002 |
| Current smoker (%) | 27 (10%) | 23 (11%) | 12 (4%) | <0.001 |
| Ex smoker (%) | 171 (61%) | 106 (52%) | 139 (51%) |  |
| Non-smoker (%) | 81 (29%) | 76 (37%) | 122 (45%) |  |
| Hypertension | 195 | 139 | 168 | 0.13 |
| Hypercholesterolaemia | 183 | 146 | 191 | 0.28 |
| CVD | 131 | 109 | 132 | 0.35 |
| Diabetes | 174 | 115 | 173 | 0.25 |
| Total cholesterol (mmol/L) | 4.40 ± 1.0 | 4.10 ± 0.97 | 4.35 ± 1.11 | 0.005 |
| LDL-Cholesterol (mmol/L) | 2.56 ± 0.88 | 2.10 ± 0.84 | 2.30 ± 0.97 | <0.001 |
| HDL-Cholesterol (mmol/L) | 1.43 ± 0.46 | 1.27 ± 0.40 | 1.40 ± 0.40 | <0.001 |
| Triglycerides (mmol/L) | 1.37 ± 0.81 | 1.66 ± 0.88 | 1.41 ± 0.40 | <0.001 |

**Figure S1A:** Box plot of difference between true distance and formula distance according to age decile in TASCFORCE (A) and SUMMIT (B) populations.

**
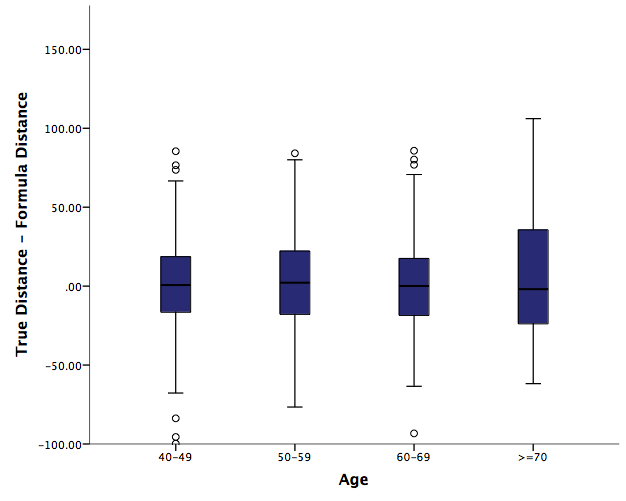
**

**Figure S1B:** Box plot of difference between true distance and formula distance according to age decile in TASCFORCE (A) and SUMMIT (B) populations.

**
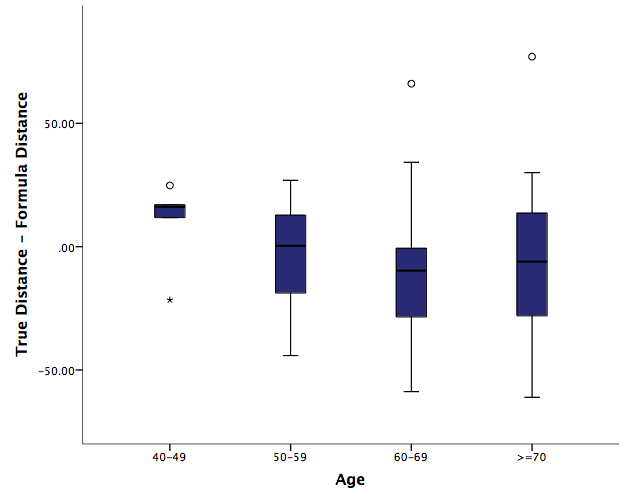
**
